# P73. Functional characterisation of HBV-specific T cell receptors for redirection of T cells against HBV infected hepatocytes

**DOI:** 10.1186/2051-1426-2-S2-P47

**Published:** 2014-03-12

**Authors:** K Krebs, K Metzger, L Weigand, C Dargel, E Kieback, W Uckert, D Busch, A Krackhardt, U Protzer

**Affiliations:** 1 TU Muenchen / Helmholtz Zentrum Muenchen, Institute of Virology, Muenchen, Germany; 2 Klinikum rechts der Isar, III. Medizinische Klinik, Muenchen, Germany; 3 Max-Delbrück Center for Molecular Medicine, Berlin, Germany; 4 TU Muenchen, Institute for Medical Microbiology Immunology and Hygiene, Muenchen, Germany

## 

Chronic HBV infection, which is accompanied by a weak and oligoclonal T cell response, is the most common cause of hepatocellular carcinoma (HCC). Current antiviral therapies do not eliminate the virus, but T cell therapy will very likely do so. From PBMCs of two HLA-A2^+^ acutely infected patients and a donor who cleared HBV infection we have established several HBV-specific monoclonal T cell lines. Thereof we isolated 11 different T cell receptors (TCR) that are specific for the HBV S-protein derived peptides S20 (FLLTRILTI) and S172 (WLSLLVPFV) or for the C18 core-peptide (FLPSDFFPSV). The aim of this study was a functional comparison of our set of HBV-specific TCRs in order to identify TCRs with optimal recognition of HBV peptides presented on HLA-A2.

By murinization and codon-optimisation of gene sequences of TCR a and b chains, fused by a P2A element for polycystronic expression, TCR expression after retroviral transduction was increased 2-fold to 60% of PBMCs expressing an HBV-specific TCR.

PBMCs transduced with the 11 optimised HBV-specific TCRs were compared in killing assays using peptide-pulsed T2 cells, LCLs and HBV-replicating HepG2.2.15 cells as targets. CD8^+^ T cells transduced with the core-specific TCRs killed target cells loaded with 0.01 nM of peptide. Cells specific for the S20 and S172 peptide were less sensitive with a specific lysis as low as 0.1 nM. Expression of most of the HLA-A2 restricted HBV-specific TCRs in CD4^+^ T cells also led to specific cytotoxicity, which was 10-fold reduced in sensitivity compared to CD8^+^ T cells and independent of CD8 co-receptor binding. Notably, our HBV-specific TCRs recognised peptide presented on various different HLA-A2 subtypes.

CD8^+^ T cells transduced with HBV-specific TCRs were also able to recognise endogenously processed peptides and specifically kill HBV-replicating hepatoma cells and strongly reduce cccDNA levels in HBV-infected HepaRG cells.

In addition, intracellular cytokine staining after stimulation showed that the TCR-transduced CD8^+^ T cells were polyfunctional, secreting INF-γ, TNF-α and IL-2, whereas CD4^+^ T cells produced mainly TNF-a and/or IL-2.

We will further analyse our HBV-specific TCRs in HBV/HLA-A2 transgenic mice in order to identify the TCR that confers best antiviral activity. Our HBV-specific TCRs may be used for elucidating specific anti-HBV mechanisms exerted by T cells, and most importantly, for adoptive T cell therapy of chronic hepatitis B and HBV-induced HCC.

